# Molecular characterization of PI^*^S_*hangzhou*_, a *SERPINA1* allele from continental China encoding a defective alpha-1-antitrypsin

**DOI:** 10.3389/fped.2022.985892

**Published:** 2022-09-14

**Authors:** José M. Hernández-Pérez, Mario A. González Carracedo, Angelines Concepción García, José A. Pérez Pérez

**Affiliations:** ^1^Department of Pneumology, La Palma General Hospital, Breña Alta, Spain; ^2^Genetics Laboratory, Institute of Tropical Diseases and Public Health of the Canary Islands (IUETSPC), University of La Laguna, San Cristóbal de La Laguna, Spain; ^3^Department of Pediatrics, La Palma General Hospital, Breña Alta, Spain

**Keywords:** alpha-1-antitrypsin, continental China, deficiency allele, liver failure, SERPINA1

## Abstract

Alpha-1-antitrypsin deficiency (AATD) is a heritable condition that predisposes to respiratory and hepatic complications. Screenings in East Asia human populations for the AATD alleles most commonly found among Caucasians have yielded poor outcomes. Serum alpha-1-antitrypsin (AAT) levels, AAT phenotypes, and sequences of *SERPINA1* gene were examined in a Chinese child with a moderate deficit of serum AAT, who had suffered several episodes of liver disease, as well as in his first-order relatives. Results allowed the identification of PI^*^S_*hangzhou*_, a novel *SERPINA1* defective allele, which has been characterized by a L276R substitution, found in a *SERPINA1-*M3 genetic background. Moreover, potential effects of PI^*^S_*hangzhou*_ mutation over the AAT structure were studied by 3D homology modeling. The presence of an arginine residue at position 276 could destabilize the tertiary structure of AAT, since it occurs at a highly conserved hydrophobic cavity in the protein surface, and very close to two positively-charged lysine residues. Attending to the frequency of R276 variant reported in databases for individuals of East Asian ancestry, the PI^*^S_*hangzhou*_ allele may explain the global prevalence of the PiS phenotype observed in China.

## Introduction

Alpha-1-antitrypsin (AAT) is a monomeric glycoprotein mainly synthetized by hepatocytes and released into the bloodstream. Serum levels of AAT normally range between 1.5 and 3.5 g/l, being the major circulating antiprotease in humans. The primary function of this serine protease inhibitor is to protect the lower respiratory tract against attack by the enzyme elastase, which is released in the lung by neutrophils during infectious and inflammatory processes. In addition to regulating other proteases, AAT is an acute-phase reactant with immunomodulatory and anti-inflammatory properties (1).

Alpha-1-antitrypsin deficiency (AATD) is an inherited condition caused by mutations in the *SERPINA1* gene that negatively affect AAT level and/or activity. AATD predisposes to lung disease in adults and liver disease during childhood or later in adult life. The high variability in the clinical manifestations of AATD suggests a strong influence of genetic and environmental factors, as exposure to cigarette smoke in the case of lung disease. Respiratory complications, typically panacinar emphysema, arise from insufficient serum AAT level for providing protection of lung tissue against damage by neutrophil elastase. In the other hand, certain missense mutations in the *SERPINA1* gene, as the PI^*^Z allele, cause the accumulation of toxic aggregates of misfolded AAT in the endoplasmic reticulum of hepatocytes, switching on a cellular stress response that may result in diverse clinical presentations as neonatal cholestasis or late-onset cirrhosis and hepatocellular carcinoma in adults (1, 2).

Hundreds of *SERPINA1* variants have been described and about 70 of them have been associated with clinical manifestations (3, 4). The most common AATD alleles among individuals of European descent are PI^*^S and PI^*^Z, with frequencies of 5–10 and 1–3%, respectively. Nearly 100 percent of the clinical cases of AATD-associated pathologies in these populations involve the PI^*^Z allele (2). However, these deficiency alleles do not have the same relevance in other human populations. For example, PI^*^Z seems to be absent in China, whereas PI^*^S is far from being a genetic polymorphism in this country (5).

## Materials and methods

A Chinese family from Hangzhou (capital of Zhejiang Province) consisting of four members, the parents and two siblings, has been examined in this study. Written informed consent was obtained from the parent in order to conduct a genetic analysis in the context of AATD. The study was declared exempted from approval by the Ethical Committee of the General Hospital of La Palma (Canary Islands, Spain), since it was circumscribed to a single family in the context of AATD. Ethical principles for medical research involving human subjects were followed as described by Declaration of Helsinki.

Plasmatic AAT levels were quantified by immunonephelometry, while AAT phenotypes were determined by isoelectric focusing (IEF) electrophoresis, following standardized clinical laboratory methods (6). Genomic DNA was extracted from dried blood samples through an alkaline lysis method (7). The complete coding exons (2–5) of *SERPINA1* gene (RefSeq NG_008290.1) and the corresponding exon-intron boundaries were sequenced using PCR primers and conditions described elsewhere (8).

To predict the effect of the non-synonymous substitution studied in the present work over the protein function, AAT sequences of 85 different mammalian species were retrieved from GenBank and aligned with Clustal Omega, at the EMBL-EBI server (9). The multiple alignment was then loaded to WebLogo (10) and ConSurf (11) servers, to analyze the residue conservation either at AAT amino-acid sequence and 3D structure, respectively, using default parameters. Conservation percentages were manually calculated for each position of the alignment. The RCSB-PDB entry 1HP7.1, which corresponds to a 2.1 Å resolution structure of mature AAT (12), was used as template to obtain a 3D homology model for the L276R variant with the Swiss Model server (13). The PyMOL graphics system (14) was finally used to visualize hydrophobicity and charge patterns over the protein surface, with the aim of Color_h and YRB scripts (15). All amino-acid positions are referred to mature AAT, after processing the 24 amino acids of the signal peptide.

## Results

**Figure 1:**
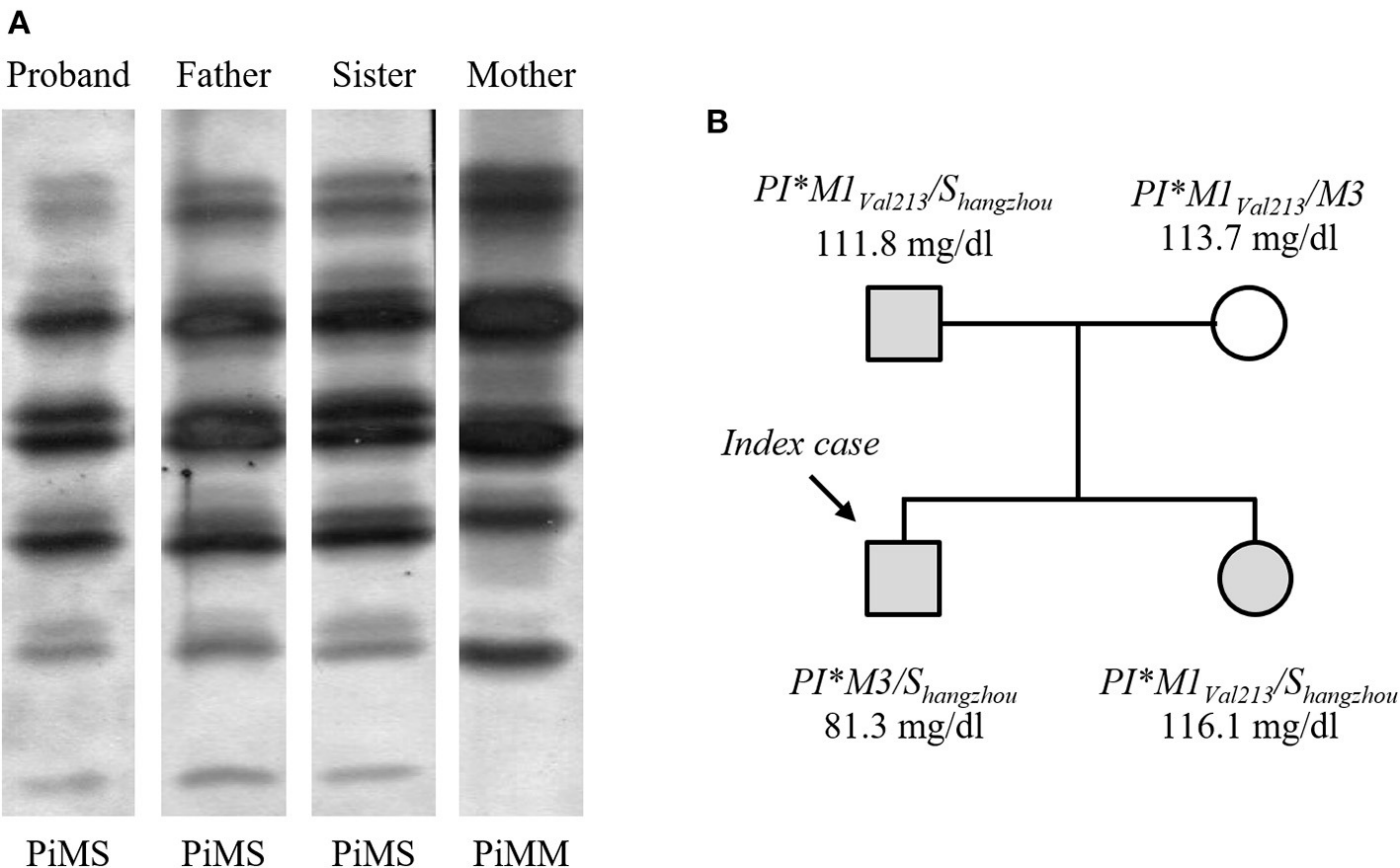
Family study of AATD. **(A)** AAT phenotypes determined by IEF analysis. **(B)** Pedigree of the proband's family showing the genotypes for *SERPINA1* gene and serum AAT levels. Carriers of the *S*_*hangzhou*_ allele are highlighted in gray.

The index case of this study was a male infant who was admitted to the hospital due to severe jaundice. His plasma levels of total bilirubin (20.4 mg/dl) and gamma-glutamyl transpeptidase (139 U/l) were elevated. Although ultrasound examination did not reveal focal lesions in liver, the biochemical evidences of hepatic damage prompted us to investigate the possibility of AATD in the patient. In this sense, IEF analysis of AAT pointed out a PiMS phenotype (Figure 1A). However, AAT concentration in the serum of the proband (81.3 mg/dl) was below the reference ranges (95% confidence intervals) described for PiMS phenotype by several authors (16). Surprisingly, molecular characterization of *SERPINA1* gene by DNA sequencing showed that the patient was homozygous for the wild-type variant of the SNP rs17580 (NM_000295.5:c.863A>T; p.Glu264Val, considering mature AAT sequence), but instead he carried a mutant variant of the multiallelic locus rs550592374, specifically the non-synonymous substitution NM_000295.5:c.899T>G (p.Leu276Arg of mature AAT) in exon 3, which was found in a *SERPINA1*-M3 allelic background. Since the birth of the index case in La Palma Island was circumstantial, and the birthplace of both parents was Hangzhou City (China), the new AATD allele was named PI^*^S_*hangzhou*_ to ensure its traceability. Diagnosis of AATD was further extended to three relatives of proband. His father and sister also showed a PiMS phenotype and both carried the PI^*^S_*hangzhou*_ allele (Figure 1B). The average serum AAT level of the three PI^*^M/S_*hangzhou*_ subjects was 103.1 mg/dl.

**Figure 2:**
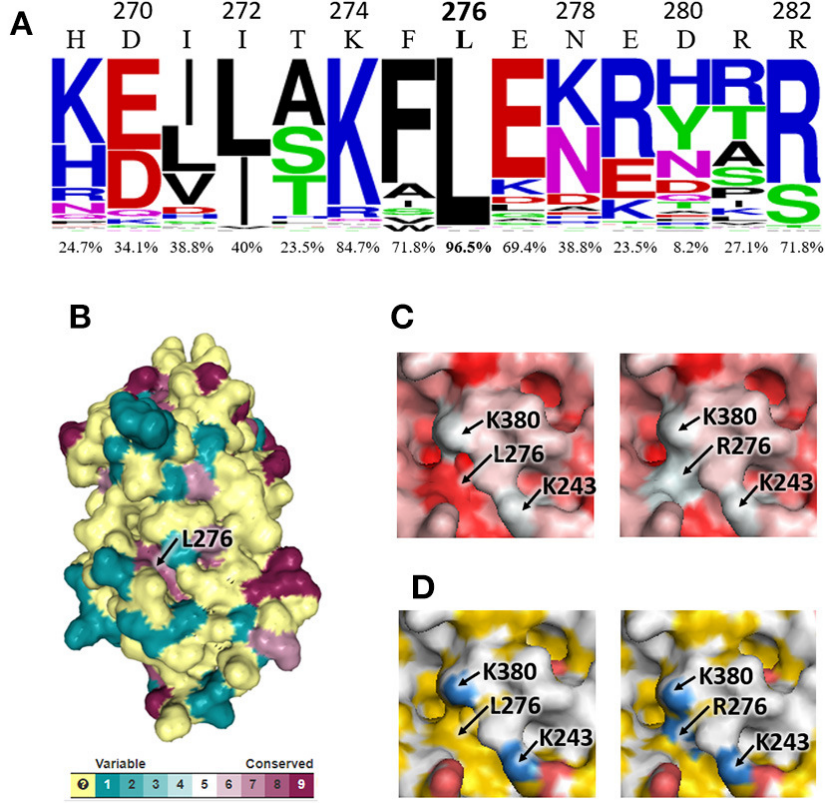
Bioinformatic analysis of the impact of L276R variant on human AAT. **(A)** WebLogo representation of the alignment region that contains the L276 residue. Numbers above each amino acid symbol indicate positions in mature human AAT, while percentages indicate the conservation level of the residues present in human AAT. **(B)** 3D model of AAT obtained with Consurf server from the multiple alignment of AAT sequences. Different colors represent conservation scores. **(C,D)** Structural details of the cavity where L276R substitutions occurs, visualized with PyMOL. Two positive charged lysine residues (K380 and K243) are also indicated. Different colors represent different chemical properties of amino acids, ranging from hydrophilic (white) to hydrophobic (red) in **(C)** and from positive-charged (blue) to neutral (yellow) and negative-charged (red) in **(D)**.

The alignment of AAT sequences from 85 mammalian species showed that leucine at position 276 is highly conserved (96.5% of the sequences), as well as adjacent residues to a lesser extent (Figure 2A). L276 was found to be exposed at the AAT surface, inside a well-conserved protein cavity (Figure 2B), and its substitution by arginine modifies the surface characteristics of the cavity, which becomes more hydrophilic and positively-charged (Figures 2C,D). It is noteworthy that in the mutant AAT encode by PI^*^S_*hangzhou*_ allele, R276 is very close to two positively-charged amino acids: K243 and, especially, K380 (Figures 2C,D). Therefore, the electrostatic repulsion between R276 and K243/K380 residues could destabilize the tertiary structure of mutant AAT. Our analysis did not detect any interaction between R276 and the acidic amino acid at position 376 that distinguishes AATs encoded by PI^*^M3 (D376) and PI^*^M1_*Val*213_ (E376) alleles.

## Discussion

The NM_000295.5:c.899T>G (p.Leu276Arg of mature AAT) substitution in the *SERPINA1* gene, present as mutant variant in the PI^*^S_*hangzhou*_ allele, was reported for the first time by the Exome Aggregation Consortium (ExAC) (17), together with the NM_000295.5:c.899T>C (p.Leu276Pro) substitution as the multiallelic SNV rs550592374. Currently, the R276 variant of AAT is included in the Genome Aggregation Database (gnomAD; v2.1.1 dataset) under the identifier 14-94847226-A-C, and has been exclusively detected in individuals of East Asian ancestry (not Japanese or Korean), with a frequency of 0.004. Interestingly, the frequency of the mutant variant V264, which characterized the classical PI^*^S allele, is nearly 60-fold lower (0.00007) than frequency of R276 in the same set of 7,212 individuals. However, a meta-analysis of epidemiological surveys estimated that the frequency of AAT alleles leading to the PiS phenotype in the Chinese population is 0.001 (6,806 subjects; 20 cohorts) (5). In conclusion, the PI^*^S_*hangzhou*_ allele may underlie the global prevalence of PiS-like phenotypes observed in China. Is should be noted that frequency of AAT alleles leading to the PiS phenotype is significantly higher in certain regions of China, as in Fujien (0.0066) (18) and Guangxi (0.0072) (19) provinces, and this may be related to the fact that two homozygous subjects are included among the 56 carriers of the R276 variant registered in the gnomAD dataset from East Asian.

With regard to the functional effects of non-synonymous substitutions on AAT, the R276 variant is classified (gnomAD) as “benign” and “tolerated” by the bioinformatic predictors Polyphen and SIFT, respectively, while the P276 variant (identifier 14-94847226-A-G in gnomAD) is categorized as “probably damaging” and “deleterious” by the same analysis tools. However, a “conflicting interpretation of pathogenicity” is stated in the ClinVar Database (NCBI) for the R276 variant. Most computational tools for predicting the impact of missense variants on protein function examine various features as evolutionary conservation and physicochemical properties of amino acids, or secondary structure of the polypeptide. In this sense, it is known that proline, but not arginine, alters the secondary structure when it replaces an amino acid residue (20, 21). Therefore, amino acid substitutions by proline are commonly considered as deleterious, while arginine substitutions can be easily predicted to be tolerated. The sensitivity in predicting the deleterious effect of amino acids substitutions is enhanced when the tertiary structure of proteins is examined (3). Our computational analysis of mutant AAT encoded by the PI^*^S_*hangzhou*_ allele indicated that L276R substitution occurs in a highly conserved hydrophobic cavity, where the arginine residue remains positively charged (21) and in close proximity to two lysine residues (K243 and K380). This could affect the conformational stability of the mutated AAT in some degree, since exposed charged residues play important roles in protein stability (22), leading to the moderate deficiency of circulating AAT observed in the three PI^*^M/S_*hangzhou*_ heterozygous. Although serum AAT concentration in these subjects is above the protective level for pulmonary emphysema (50 mg/dl) (2), this could not be the case in subjects homozygous for PI^*^S_*hangzhou*_ allele. Additionally, the potential misfolding and aggregation of the mutated AAT within hepatocytes may be involved in the several episodes of liver complications suffered by the index case. Verification of this hypothesis requires further analysis of the molecular pathophysiology of PI^*^S_*hangzhou*_ allele, ideally by *in vivo* studies with homozygous individuals. In this sense, it should be considered that L276R substitution in the PI^*^S_*hangzhou*_ allele is found in the *SERPINA1*-M3 genetic background, and that the AAT encoded by PI^*^M3 allele has recently been described as having the ability to form aggregates like AAT encoded by PI^*^Z allele (23). This susceptibility may be increased by the presence of an arginine residue at position 276, as the proband showed lower AAT levels than his father/sister, which also carries the PI^*^S_*hangzhou*_ allele, but in combination with PI^*^M1 Nevertheless, another possible explanation of the lower AAT levels found in the proband is that pediatric population seems to have lower AAT levels than adults (24).

In conclusion, the PI^*^S_*hangzhou*_ allele has been characterized in a neonatal patient who has suffered several episodes of severe jaundice, and with reduced serum AAT level. The novel AAT variant (R276) is different from the classic PI^*^S allele (V264), but also confers a PiS phenotype. Therefore, future validation of our findings in independent study populations of Chinese ancestry, including larger sample sizes, would be very interesting. Bioinformatic analysis of mutant AAT 3D structure provides a possible explanation for AAT deficiency. However, functional investigation of this mutation is required to better understand the underlying molecular and cellular mechanisms. Finally, based on available genetic and phenotypic frequency data, we conclude that PI^*^S_*hangzhou*_ allele may underlie the global prevalence of the PiS phenotype observed in China.

## Data availability statement

The data presented in the study are deposited in the GenBank repository (https://www.ncbi.nlm.nih.gov/genbank), accession number OP346042.

## Ethics statement

Ethical review and approval was not required for the study on human participants in accordance with the local legislation and institutional requirements. Written informed consent to participate in this study was provided by the participants' legal guardian/next of kin. Written informed consent was obtained from the individual(s), and minor(s)' legal guardian/next of kin, for the publication of any potentially identifiable images or data included in this article.

## Author contributions

JH-P: conception and design of the study, collection of patient samples and clinical data, analysis and interpretation of data, and writing and discussion of the article. MG: genotyping of the *SERPINA1* gene, analysis and interpretation of data, and discussion of the article. AG: collection of newborn sample and clinical data and discussion of the article. JP: conception and design of the study, genotyping of the *SERPINA1* gene, analysis and interpretation of data, and writing and discussion of the article. All authors contributed to the article and approved the submitted version.

## Conflict of interest

The authors declare that the research was conducted in the absence of any commercial or financial relationships that could be construed as a potential conflict of interest. The reviewer BM-D declared a past co-authorship with one of the authors JP to the handling editor.

## Publisher's note

All claims expressed in this article are solely those of the authors and do not necessarily represent those of their affiliated organizations, or those of the publisher, the editors and the reviewers. Any product that may be evaluated in this article, or claim that may be made by its manufacturer, is not guaranteed or endorsed by the publisher.
